# The Vertical and Horizontal Relations of Korean and Korean American Older Adults and Their Well-Being

**DOI:** 10.1093/geroni/igab046.1171

**Published:** 2021-12-17

**Authors:** Meeryoung Kim, Nan Sook Park, Michin Hong

## Abstract

Various relationships are important for the well-being of older adults. This session focuses on the vertical and horizontal relations of Korean and Korean American older adults and their well-being. The purpose of this session is to highlight the importance of intergenerational relations and social involvement of Korean and Korean American older adults. For vertical relations, two studies focus on intergenerational relationships and solidarity. The first study investigated whether intergenerational relationships and social support mediate the distressing consequences of life events, and how this improved the psychological well-being of Korean older adults. The second study developed a standardized measurement tool for intergenerational solidarity because intergenerational conflicts caused by rapid socioeconomic changes have highlighted the importance of strengthening intergenerational solidarity. The third and fourth studies focus on horizontal relations involving social isolation and social involvement. Guided by the double jeopardy hypothesis, the third study examined the health risks posed by the coexistence of social and linguistic isolation in older Korean Americans. As the opposite of social isolation, social involvement was an important factor of social integration of older adults. The fourth study examined volunteering as an example of social involvement by focusing on older adults’ volunteering on the social integration and role identity. Implications of this study suggest not only the importance of social involvement but also the intergenerational relationships on older adults’ well-being.

